# 

**Published:** 1893-10

**Authors:** 


### CONTENTS FOR OCTOBER, 1893.

ORIGINAL ADDRESS. PAGE.

School-life and Physical Development. By Louis A. Weigel, M. D. .... 129

ORIGINAL COMMUNICATIONS.

Intubation. By George F. Cott, M. D. .... 137
The Treatment of Pott's Disease of the Spine. By A. B. Judson, M. D. .... 143
A Review of the Operative Treatment for the Radical Cure of Inguinal Hernia. By Samuel E. Milliken, M. D. .... 146

CLINICAL REPORT.

Cysts of the Nasal Passages. By W. S. Renner, M. D. .... 150

PROGRESS IN MEDICAL SCIENCE.

Nervous Diseases. By James W. Putnam, M. D. .... 154

SELECTIONS.

Superficial Laceration of the Female Perineum .... 157
The Causation of Anemia and the Blood-Changes Produced by Uric Acid .... 158
Dislocation of the Cervical Vertebra .... 159
The Treatment of Hemotysis .... 160
Academy of Medicine Notes .... 161

EDITORIAL.

The First Pan-American Medical Congress, .... 162
The Relation of Noises to Health Again .... 164
Notes of the Congress .... 168
Personal—Obituary .... 171
Society Meetings .... 173
Book Reviews .... 177
Literary Notes .... 188

